# Silencing of Keratinocyte Growth Factor Receptor Restores 5-Fluorouracil and Tamoxifen Efficacy on Responsive Cancer Cells

**DOI:** 10.1371/journal.pone.0002528

**Published:** 2008-06-25

**Authors:** Sabrina Rotolo, Simona Ceccarelli, Ferdinando Romano, Luigi Frati, Cinzia Marchese, Antonio Angeloni

**Affiliations:** 1 Dipartimento di Medicina Sperimentale, Università Sapienza di Roma, Roma, Italy; 2 Istituto Neurologico Mediterraneo “Neuromed”, Pozzilli, Italy; Lehigh University, United States of America

## Abstract

**Background:**

Keratinocyte growth factor receptor (KGFR) is a splice variant of the FGFR2 gene expressed in epithelial cells. Activation of KGFR is a key factor in the regulation of physiological processes in epithelial cells such as proliferation, differentiation and wound healing. Alterations of KGFR signaling have been linked to the pathogenesis of different epithelial tumors. It has been also hypothesized that its specific ligand, KGF, might contribute to the development of resistance to 5-fluorouracil (5-FU) in epithelial cancers and tamoxifen in estrogen-positive breast cancers.

**Methodology/Principal Findings:**

Small interfering RNA was transfected into a human keratinocyte cell line (HaCaT), a breast cancer derived cell line (MCF-7) and a keratinocyte primary culture (KCs) to induce selective downregulation of KGFR expression. A strong and highly specific reduction of KGFR expression was observed at both RNA (reduction = 75.7%, *P* = 0.009) and protein level. KGFR silenced cells showed a reduced responsiveness to KGF treatment as assessed by measuring proliferation rate (14.2% versus 39.0% of the control cells, *P*<0.001) and cell migration (24.6% versus 96.4% of the control cells, *P* = 0.009). In mock-transfected MCF-7 cells, KGF counteracts the capacity of 5-FU to inhibit cell proliferation, whereas in KGFR silenced cells KGF weakly interferes with 5-FU antiproliferative effect (11.2% versus 28.4% of the control cells, *P* = 0.002). The capacity of 5-FU to induce cell death is abrogated by co-treatment with KGF, whereas in KGFR silenced cells 5-FU efficiently induces cell death even combined to KGF, as determined by evaluating cell viability. Similarly, the capacity of tamoxifen to inhibit MCF-7 and KCs proliferation is highly reduced by KGF treatment and is completely restored in KGFR silenced cells (12.3% versus 45.5% of the control cells, *P*<0.001).

**Conclusions/Significance:**

These findings suggest that selective inhibition of the KGF/KGFR pathway may provide a useful tool to ameliorate the efficacy of the therapeutic strategies for certain epithelial tumors.

## Introduction

Keratinocyte growth factor receptor (KGFR/FGFR2-IIIb) is a tyrosine kinase protein that belongs to the family of the fibroblast growth factor receptors (FGFRs). KGFR represents a splicing transcript variant of FGFR2 gene and is expressed on epithelial cells of different organs. The alternatively spliced isoform, known as FGFR2-IIIc, is found in cells of mesenchymal lineages [1], [2]. KGFR plays a key role in the control of epithelial growth and differentiation, carrying out its biological effects in a paracrine way [3] through high affinity binding to its specific ligands, namely keratinocyte growth factor (KGF/FGF7), FGF10 and FGF22 [4]. Among them, KGF acts not only as a potent mitogen for primary human keratinocytes, but also promoting their differentiation program [5] and protecting them against apoptosis induction [6], [7]. Furthermore, KGF is involved in both experimental [8], [9] and *in vivo* [10] wound healing models, stimulating migration of keratinocytes [11], [12] and inducing reorganization of actin cytoskeleton, therefore increasing epithelial cell motility [13].

Recently, there has been growing interest about the potential role of alterations of KGF/KGFR signaling in epithelial tumorigenesis. Increased KGFR mRNA expression has been detected in a wide range of tumors of epithelial origin, such as lung, colon, gastric, pancreas and prostate cancers. In some cases, such increased expression seems to be associated with cell transformation and, perhaps, malignant progression [14]. Moreover, KGF administration has been shown to increase cell motility in estrogen receptor (ER)-positive breast tumor cells [15], [16], to be potentially involved in breast cancer progression and metastasis [17] and to enhance the invasive potential of gastric carcinoma derived cell lines overexpressing KGFR [18].

Other studies led to hypothesize that KGF may exert antiapoptotic activity on certain cancer cells as well as inhibition of apoptosis induced by the chemotherapeutic drug 5-fluorouracil (5-FU) [19]–[22]. The development by cancer cells of resistance to traditional chemotherapeutic agents, such as 5-FU, is frequently observed and remains a major obstacle to a successful treatment of cancer and a prominent cause of tumor recurrence after chemotherapy [23].

Furthermore, it has been suggested that alterations of the pathways involving FGFs and cognate receptors might represent one of the mechanisms of resistance to tamoxifen that finally develops in many ER-positive breast cancers [24]–[27]. However, this hypothesis is not completely ascertained and the specific role played by the large family of the FGFs is far from being understood.

The approach based on selective downregulation of proteins involved in cellular processes correlated to tumor progression represents a promising frontier for cancer treatment. Transfection of specific small interfering RNAs (siRNAs) is a powerful tool to achieve a gene-specific knockdown and represents a potent therapeutic strategy for the treatment of several diseases, such as viral infections, neurological disorders and cancers [28].

The exclusive target specificity of siRNA against disease-relevant mRNAs is an essential prerequisite for utilization of this technology. In this study, we selectively downregulated KGFR mRNA and protein expression in three epithelial cell lines, HaCaT keratinocytes, MCF-7 breast cancer cell and primary cultured keratinocytes (KCs), by a new approach of siRNA design, based on the utilization of DICER endonuclease substrate 27-mer dsRNAs to trigger RNA interference (RNAi). This technique provides enhanced efficacy and longer duration of RNAi as compared to traditional 21-mer siRNAs, allowing usage of lower concentrations of RNAi in target cells, which greatly reduces the side effects. Furthermore, it allows the targeting of sites that are refractory to suppression with 21-mer siRNAs [29].

We analyzed the effects of KGFR siRNA on characteristics bio-parameters such as cell viability, proliferation, apoptosis and migration of the tested cell lines. Finally, we evaluated whether the downregulation of KGFR expression is able to inhibit 5-FU and tamoxifen resistance induced by KGF in cell cultures.

## Results

### Inhibition of KGFR mRNA expression by siRNAs

**Figure 1:**
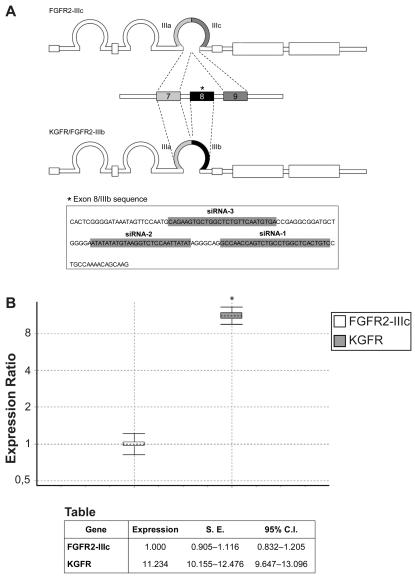
KGFR and FGFR2-IIIc mRNA relative expression levels in MCF-7 cells. (A) Schematic drawing representing the alternative splicing of FGFR2-IIIc and KGFR/FGFR2-IIIb. The inset (*) reports the cDNA sequence (nucleotides from 1590 to 1698) corresponding to the whole exon 8, with the sequences targeted by the three siRNAs (grey boxes). (B) The levels of KGFR and FGFR2-IIIc mRNA expression were determined by Q-RT-PCR, and normalized to β-actin mRNA levels. KGFR mRNA in MCF-7 cells was determined as fold with respect to FGFR2-IIIc mRNA. The graph show the interquartile range of three independent experiments (boxes), their mean (horizontal dotted bars) and 95% Confidence Interval (CI) (whiskers). Two-sided Student's *t* test was used to compare KGFR versus FGFR2-IIIc expression: * *P*<0.001. The accompanying table reports mean expression level, Standard Error (S.E.), and 95% CI for each assayed sample.

**Figure 2:**
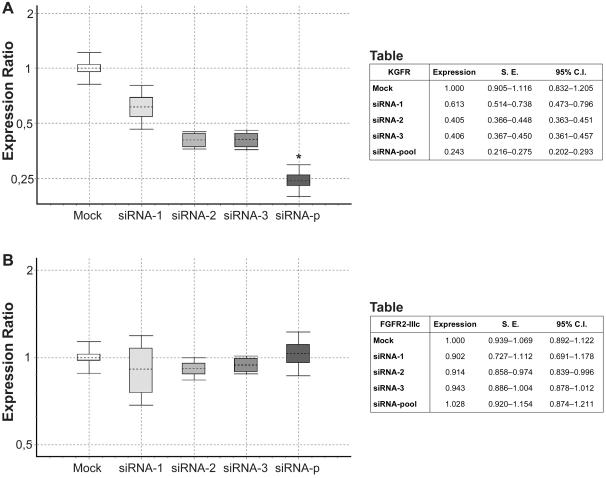
Effect of three selected siRNAs on KGFR and FGFR2-IIIc mRNA expression levels. (A, B) MCF-7 cells were mock transfected or transfected with 5 nM siRNA-1, -2, -3, or with a pooled set of them, and total RNA was extracted 48 h after transfection. The levels of KGFR (A) and FGFR2-IIIc (B) mRNA expression were determined by Q-RT-PCR, and normalized to β-actin mRNA levels. KGFR (A) or FGFR2-IIIc (B) mRNA in siRNA-transfected cells were determined as fold with respect to that expressed in mock-transfected cells. For each treatment, the graphs show the interquartile range of three independent experiments (boxes), their mean (horizontal dotted bars) and 95% Confidence Interval (CI) (whiskers). Two-sided Student's *t* test was used to compare siKGFR-transfected versus mock-transfected cells: * *P* = 0.009. The accompanying tables report mean expression level, Standard Error (S.E.), and 95% CI for each assayed sample.

There are no clear rules governing siRNA target site selection for specific mRNA sequences. However, within a single mRNA sequence different siRNA molecules show a dramatic variability in terms of efficacy and specificity of gene silencing. Here we used laboratory- and web-based programs, according to the previously described criteria (http://www.rockefeller.edu/labheads/tuschl/sirna.html), to select three siRNAs sequences directed against the FGFR2 gene. It is known that the same gene codes for two alternative transcripts, designated as KGFR/FGFR2-IIIb and FGFR2-IIIc, that differ for a divergent stretch of 49 amino acids in their extracellular domain and display different ligand-binding characteristics. Thus, to realize a specific knockdown of the KGFR transcript, we selected siRNAs sequences targeted within the exon 8 of FGFR2 gene, which is spliced only in the KGFR isoform (Fig. 1A). All the siRNAs sequences were entered into a BLAST search to ensure that there was no significant homology with other genes. As concerning the relative expression of the two FGFR2 isoforms in our experimental models, HaCaT cells have been previously shown to express the FGFR2-IIIc variant of FGFR2 in two orders of magnitude lesser amount than the FGFR2-IIIb splice variant [30]; in MCF-7 cells, previously shown to express both isoforms [31], we demonstrated that the FGFR2-IIIb expression is greatly higher than that of FGFR2-IIIc (11 fold increase) (Fig. 1B). The ability of each designed siRNA and of a pooled set of the three duplexes to specifically reduce the levels of KGFR mRNA, without affecting the expression of the FGFR2-IIIc isoform, was assayed in MCF-7 and HaCaT cells. Transient transfections were used to deliver each siRNA and cells incubated with the liposomal vector alone (mock transfection) were used as control. The optimal concentration for siRNA transfection was experimentally determined to be 5 nM (data not shown), in keeping with recent observations that indicated toxic mechanisms, off-target effects and stimulation of immune response induced by high doses of synthetic RNAi *in vitro* and *in vivo* [32]. The ability of the various siRNAs to reduce the amount of KGFR mRNA was estimated by Q-RT-PCR. In MCF-7 cells we also evaluated the specificity of the designed siRNAs. KGFR and FGFR2-IIIc mRNA levels were normalized to the β-actin mRNA levels. 48 h after transfection, MCF-7 cells transfected with the pooled set of siRNA-1, -2 and -3 expressed a statistically significant reduced amount of KGFR mRNA compared to the mock-transfected cells (0.243 fold, reduction = 75.7%, *P* = 0.009) (Fig. 2A). A less evident effect was obtained by transfection with the individual duplexes: siRNA-1 (0.613 fold, reduction = 38.7%, *P* = 0.168), siRNA-2 (0.405 fold, reduction = 59.5%, *P* = 0.068) and siRNA-3 (0.406 fold, reduction = 59.4%, *P* = 0.061) (Fig. 2A). Conversely, neither individual siRNAs nor the siRNA-pool showed any inhibitory effect on FGFR2-IIIc mRNA levels (siRNA-1: 0.902 fold, reduction = 9.8%, *P* = 0.71; siRNA-3: 0.914 fold, reduction = 8.6%, *P* = 0.36; si-RNA-3: 0.943 fold, reduction = 5.7%, *P* = 0.52; siRNA-pool: 1.028 fold, difference = 2.8%, *P* = 0.85) (Fig. 2B).

Therefore, all the subsequent experiments were carried out by transfecting the siRNA-pool, which matches the commonly adopted criteria of siRNA efficiency (>70% reduction in target mRNA). Furthermore, since the pool shows a high specificity for KGFR isoform, it will be referred as siKGFR in the rest of the manuscript.

### Time course of siRNA-mediated KGFR silencing

**Figure 3:**
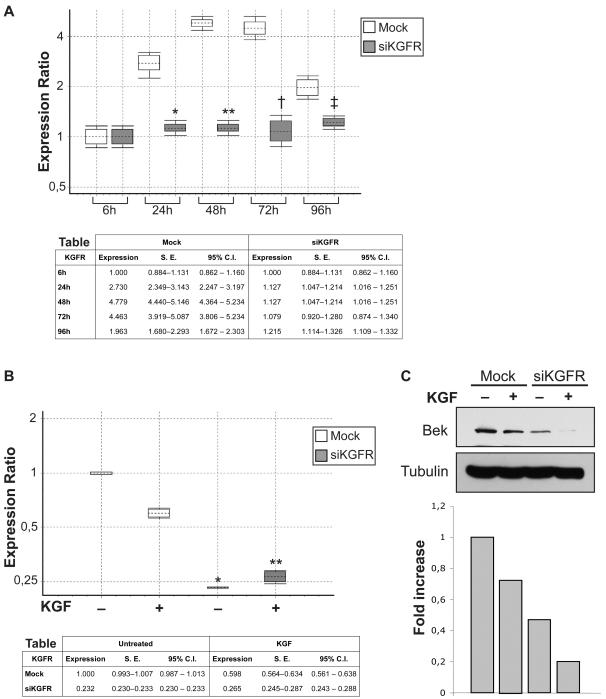
Effect of siKGFR transfection on KGFR mRNA and protein expression levels. (A) HaCaT cells were mock transfected or transfected with 5 nM siKGFR, and total RNA was extracted from the transfected cells 6, 24, 48, 72 and 96 h later. The levels of KGFR mRNA expression were determined by Q-RT-PCR, and normalized to β-actin mRNA levels. The amount of KGFR mRNA at each time point was expressed as fold of KGFR mRNA level with respect to the 6 h time point. For each time point, the graph shows the interquartile range of three independent experiments (boxes), their mean (horizontal dotted bars) and 95% CI (whiskers). The accompanying table reports mean expression level, Standard Error (S.E.), and 95% CI for each assayed sample. Two-sided Student's *t* test was used to compare siKGFR-transfected versus mock-transfected cells: * *P*<0.001 (24 h); ** *P*<0.001 (48 h); † *P*<0.001 (72 h); ‡ *P* = 0.017 (96 h). (B) HaCaT cells were mock transfected or transfected with 5 nM siKGFR. 24 h after transfection, cells were treated with 20 ng/ml KGF for 48 h. The levels of KGFR mRNA expression were determined by Q-RT-PCR, and normalized to β-actin mRNA levels. The amount of KGFR mRNA was expressed as fold of KGFR mRNA levels with respect to untreated mock-transfected cells. Graph and table report the same sets of data as in (A) for each assayed sample. *P* values were determined using two-sided Student's *t* test: * *P* = 0.003 versus untreated mock-transfected cells; ** *P* = 0.018 versus KGF-treated mock-transfected cells. (C) HaCaT cells were transfected and treated as in (B), and the levels of KGFR protein expression were determined by Western blot analysis using a polyclonal anti-bek antibody. The same blot was probed for tubulin as control for equal loading. The amount of KGFR protein was evaluated by densitometric analysis; the values from a representative experiment were standardized to tubulin levels, expressed as fold of KGFR protein with respect to untreated mock-transfected cells and reported as a graph.

To evaluate the duration and efficacy of KGFR silencing, we performed time course experiments on HaCaT cells transfected with siKGFR, determining the amount of KGFR mRNA by Q-RT-PCR at 6, 24, 48, 72 and 96 h after transfection (Fig. 3A). A reduction of KGFR mRNA expression was observed 24 h after transfection, as compared to mock-transfected cells (1.127 versus 2.730 fold, difference = 58.7%, *P*<0.001), while full efficacy was achieved at 48 and 72 h (1.127 versus 4.779 fold, difference = 76.4%, *P*<0.001 and 1.079 versus 4.463 fold, difference = 75.8%, *P*<0.001, respectively) from transfection. 96 h after transfection, a strong decrease in KGFR expression was observed also in the control. However, in transfected cells, downregulation of KGFR expression was still significant (1.215 versus 1.963 fold, difference = 38.1%, *P* = 0.017). On the bases of the time course results and according to previous reports showing that the peak of KGFR protein expression is reached at 72 h after starvation [30] we decided to perform all the subsequent experiments at 72 h following siKGFR transfection.

### Downregulation of KGFR mRNA and protein expression by siRNA in KGF-treated HaCaT cells

It is known that treatment of epithelial cells with KGF induces several events such as cell proliferation and differentiation through binding to KGFR and internalization of the receptor coupled to reduction of the level of KGFR mRNA expression. Thus, we examined the effect of siKGFR transfection in cell cultures in presence of 20 ng/ml KGF. mRNA and protein expression levels were tested by Q-RT-PCR and Western blot analysis. A sharp decrease of KGFR mRNA was observed in cells transfected with siKGFR as compared to mock-transfected cells (0.232 fold, reduction = 76.8%, *P* = 0.003) (Fig. 3B). In presence of KGF, we observed a downmodulation in the expression of KGFR also in mock-transfected cells. However, KGFR mRNA inhibition by siRNA was still evident (0.265 versus 0.598 fold, reduction = 55.7%, *P* = 0.018) (Fig. 3B). In the same set of experiments, the levels of KGFR protein expression were also assayed by Western blot. As shown in Fig. 3C, KGFR expression was significantly decreased in siKGFR-transfected cells as compared to mock-transfected cells. Densitometric analysis confirmed that siKGFR reduced protein expression, in both untreated and KGF-treated cells, by more than 50% with respect to mock-transfected cells (0.47 fold versus 1 fold, reduction = 53% and 0.20 versus 0.73 fold, reduction = 72.6%, respectively) (Graph Fig. 3C).

### siRNA-mediated downregulation of KGFR inhibits cell proliferation and cell migration induced by KGF

**Figure 4:**
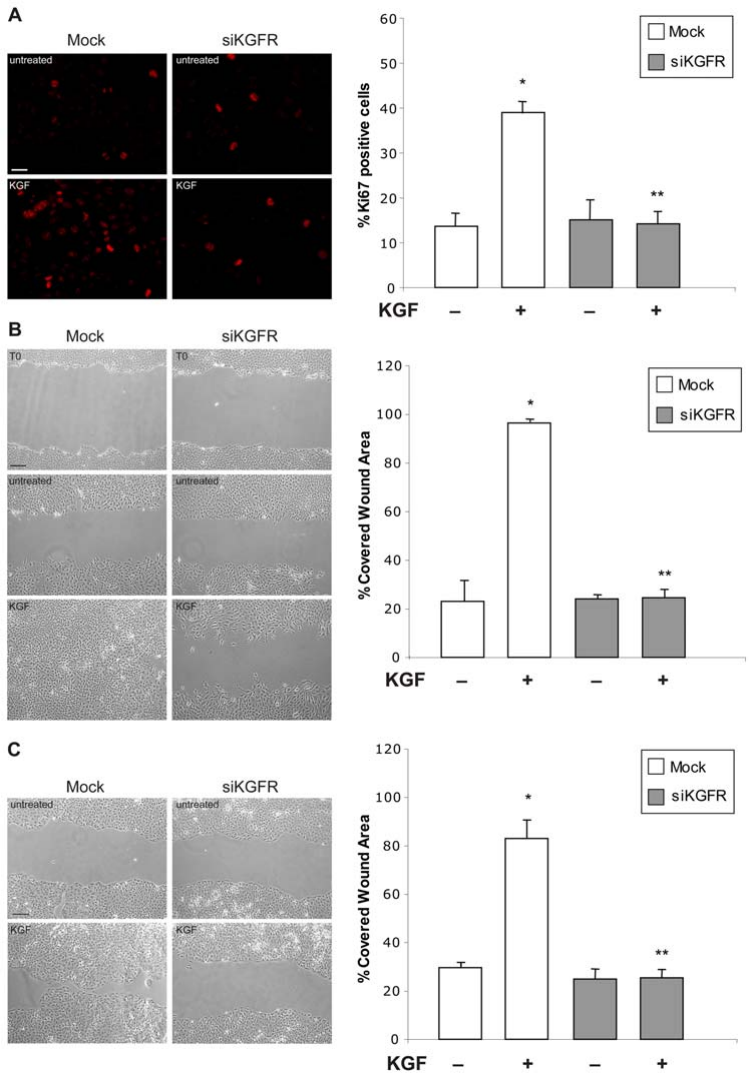
Effect of siKGFR transfection on KGF-induced proliferation and migration. (A) Proliferation assay. HaCaT cells, grown on coverslips, were mock transfected or transfected with 5 nM siKGFR. 24 h after transfection, cells were treated with 20 ng/ml KGF for 48 h. Then, cells were fixed and subjected to immunofluorescence analysis with a polyclonal antibody directed against Ki67 (red). The images are representative of three independent experiments. Scale bar, 10 µm. Nuclei were visualized using 4′, 6-diamido-2-phenylindole dihydrochloride (DAPI). The percentage of Ki67-positive cells was determined by counting the number of Ki67-positive nuclei versus total number of nuclei in ten different areas randomly taken from three different experiments, expressed as mean value±95% CI and reported as a graph. *P* values were determined using the Student's *t* test: * *P*<0.001 versus untreated mock-transfected cells; ** *P*<0.001 versus KGF-treated mock-transfected cells. (B, C) Wound-healing assay. HaCaT (B) and MCF-7 (C) cells were transfected as in (A). 48 h after transfection, a cell-free area (wound) was introduced in confluent cultures, as described in Materials and Methods. Cells were treated or not with 50 ng/ml KGF and allowed to migrate for 24 h before photography under phase contrast microscopy. The images are representative of three independent experiments. Scale bars, 10 µm. Cell migration was evaluated by repopulation of the original wound with cells: the percentage of recovered area was measured by image analysis and values in the graphs are the average of three plates for each condition±95% CI. *P* values were determined using Student's *t* test: (B) * *P*<0.001 versus untreated mock-transfected cells; ** *P*<0.001 versus KGF-treated mock-transfected cells. (C) * *P* = 0.035 versus untreated mock-transfected cells; ** *P* = 0.009 versus KGF-treated mock-transfected cells.

To evaluate the biological effects of KGFR silencing, we examined the siKGFR capacity to affect the KGF-induced proliferation by carrying out a proliferation assay on the HaCaT cell line. Plated cells were transfected with siKGFR and grown for 48 h in standard medium supplemented or not with 20 ng/ml KGF. Cell proliferation was determined by counting cells positive for Ki67 antigen, which identifies cycling cells, and reported in graph as percentage of positive cells (Fig. 4A). As expected, in mock-transfected cells we observed an increase in HaCaT cells proliferation after KGF treatment, as compared to untreated cells (39% versus 13.6%, 2.9 fold increase, *P*<0.001). The downregulation of KGFR expression through specific siRNA almost completely abolished KGF effect on HaCaT cells proliferation, with a proliferation rate to background level (14.2% versus 39%, 2.7 fold difference, *P*<0.001) (Graph Fig. 4A).

We then analyzed the impact of KGFR silencing on cell migration, another complex and strictly regulated cellular process strongly induced by KGF treatment. To this end, we performed a wound-healing assay. 48 h after transfection with siKGFR a cell-free area was introduced in monolayers of HaCaT cells, as previously described [33]. Cells treated or not with 50 ng/ml KGF, the concentration reported to be more efficient in this assay [34], were allowed to migrate from the edge of the wound for 24 h. As shown in Fig. 4B, the wound-closure was nearly completed 24 h after initial wounding in KGF-treated mock-transfected cells, while untreated mock-transfected cells showed a limited migration with respect to T0 (96.4% versus 23.1%, 4.2 fold increase, *P* = 0.035) (Graph Fig. 4B). The transfection with siKGFR significantly inhibited cell migration induced by KGF (Fig. 4B) and subsequently reduced the recovered area from 96.4% of the control cells to 24.6%, 3.9 fold difference, *P* = 0.009) (Graph Fig. 4B).

The same wound-healing assay was also performed on MCF-7 breast cancer cells, known to be responsive to KGF in terms of motility [15], with similar results (Fig. 4C). KGF treatment promoted a strong repopulation, as compared to that of untreated mock-transfected cells (83% versus 29.6%, 2.8 fold increase, *P*<0.001). In KGFR silenced cells, KGF-induced migration was nearly abolished, in comparison to control cells (25.4% versus 83%, 3.3 fold difference, *P*<0.001) (Graph Fig. 4C).

These results suggested that KGFR silencing is effective in inhibiting KGF biological effects, such as the stimulation of cell proliferation and migration, either in HaCaT keratinocytes or breast cancer epithelial cells.

### KGFR silencing inhibits the restoration of cell proliferation induced by KGF upon 5-FU stimulation

Frequently, cancer cells develop resistance to common chemotherapeutic drugs, such as 5-FU, thus challenging chemotherapy efficacy [23]. In order to investigate the role of KGFR in the establishment of resistance to 5-FU, we transiently knocked down KGFR expression by siRNA in the MCF-7 breast cancer cell line.

The transfection with siKGFR was performed in the presence or not of 20 ng/ml KGF, 25 µg/ml 5-FU or a combination of them. First, we evaluated the efficiency of KGFR silencing by analyzing both mRNA and protein expression levels.

**Figure 5:**
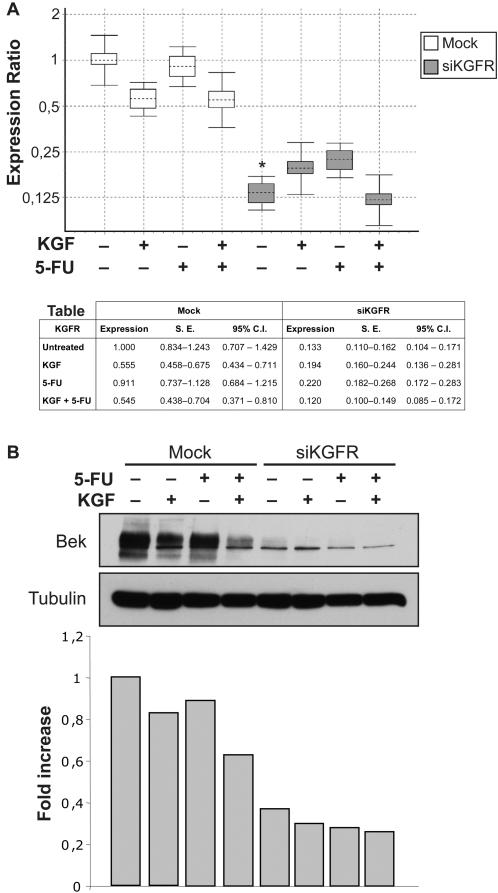
Effect of siKGFR transfection on KGFR mRNA and protein in cells treated with 5-FU. (A) MCF-7 cells were mock transfected or transfected with 5 nM siKGFR. 48 h after transfection, cells were treated with 20 ng/ml KGF, 25 µg/ml 5-FU or KGF plus 5-FU for 24 h. The levels of KGFR mRNA expression were determined by Q-RT-PCR, and normalized to β-actin mRNA levels. The amount of KGFR mRNA in siKGFR-transfected cells was expressed as fold of the level of KGFR mRNA with respect to untreated mock-transfected cells. For each treatment, the graph shows the interquartile range of three independent experiments (boxes), their mean (horizontal dotted bars), and 95% CI (whiskers). The accompanying table reports mean expression level, Standard Error (S.E.), and 95% CI for each assayed sample. *P* values were determined using the Student's *t* test: * *P* = 0.039 versus untreated mock-transfected cells. (B) MCF-7 cells were transfected and treated as in (A), and the amount of KGFR protein was evaluated by Western blot analysis with an anti-bek polyclonal antibody. Tubulin served as a loading control. The amount of KGFR protein was evaluated by densitometric analysis: the values from a representative experiment were standardized to tubulin levels, expressed as fold of KGFR level with respect to untreated mock-transfected cells and reported as a graph.

As shown in Fig. 5A, treatment with KGF of mock-transfected cells reduced the expression of KGFR mRNA compared to untreated cells, independently from the presence of 5-FU in the cell cultures (0.555 fold, reduction = 44.5%, *P* = 0.170, and 0.545 fold, reduction = 45.5%, *P* = 0.183, respectively). Moreover, 5-FU alone poorly affected KGFR mRNA expression (0.911 fold, reduction = 8.9%, *P* = 0.711). In siKGFR-transfected cultures, we observed a strong effect on mRNA expression (0.133 fold, reduction = 86.7%, *P* = 0.039), with modest variations in response to the presence or absence of KGF and/or 5-FU. These data confirmed that MCF-7 cells react similarly to HaCaT cells in response to KGFR silencing. Furthermore, the results obtained at RNA level were confirmed by analyzing protein expression, as shown in Fig. 5B. The specific siRNA significantly decreased KGFR protein in untreated cells, as well in KGF and/or 5-FU-treated cells, by 60%–70% with respect to the same treatment in mock-transfected cells (Graph Fig. 5B).

**Figure 6:**
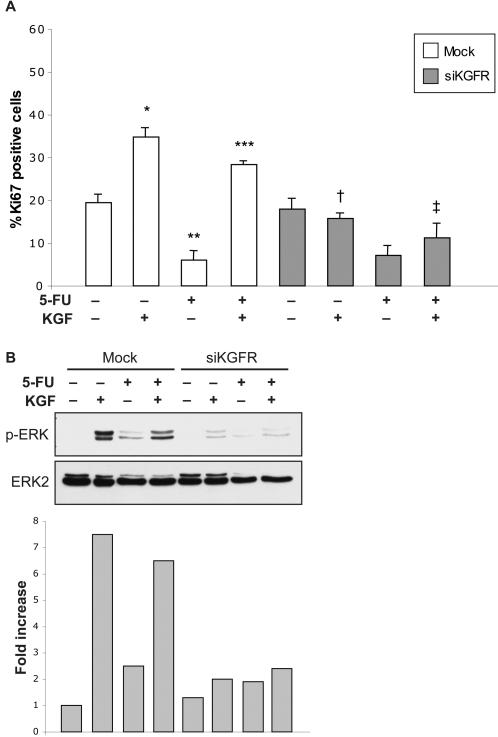
Effect of siKGFR transfection on KGF-induced inhibition of 5-FU antiproliferative activity. (A) MCF-7 cells, grown on coverslips, were mock transfected or transfected with 5 nM siKGFR and 48 h later they were treated or not with 20 ng/ml KGF, 25 µg/ml 5-FU or KGF plus 5-FU. After 24 h, cells were fixed and subjected to immunofluorescence analysis with an anti-Ki67 polyclonal antibody. Nuclei were visualized using 4′, 6-diamido-2-phenylindole dihydrochloride (DAPI). Cell proliferation was evaluated as percentage of Ki67-positive nuclei versus total number of nuclei in ten different areas randomly taken from three different experiments, expressed as mean value±95% CI and reported as a graph. *P* values were determined using the Student's *t* test: * *P* = 0.004 and ** *P*<0.001 versus untreated mock-transfected cells; *** *P*<0.001 versus 5-FU-treated mock-transfected cells; † *P*<0.001 versus KGF-treated mock-transfected cells; ‡ *P* = 0.002 versus KGF plus 5-FU-treated mock-transfected cells. (B) MCF-7 cells were transfected and treated as in (A), and Western blot analysis of the phosphorylation status of ERK was carried out using a phospho-specific ERK monoclonal antibody (p-ERK). Levels of total ERK were assessed by blotting with an ERK2-specific antibody. The amount of activated ERK was evaluated by densitometric analysis: the values from a representative experiment were standardized to total ERK levels, expressed as fold of p-ERK expression with respect to untreated mock-transfected cells and reported as a graph.

We next performed a proliferation assay on MCF-7 cells, transfected with siKGFR or mock-transfected and grown for 48 h in standard medium supplemented or not with KGF, 5-FU or a combination of them, as above. Cell proliferation was evaluated by counting cells positive for Ki67 antigen, and reported in graph as percentage of positive cells (Fig. 6A). Also in MCF-7 we found an increase in cell proliferation after KGF treatment, as compared to untreated cells (34.8% versus 19.5%, 1.8 fold increase, *P* = 0.004). As expected, treatment with 5-FU revealed an antiproliferative effect (6.0% versus 19.5%, 3.3 fold difference, *P*<0.001), which was almost completely abrogated by co-treatment with KGF (28.4% versus 6.0%, 4.7 fold difference, *P*<0.001). KGFR downregulation by siRNA nearly abolished KGF proliferative effect, both alone and in combination with 5-FU (15.7% versus 34.8%, 2.2 fold difference, *P*<0.001 and 11.2% versus 28.4%, 2.5 fold difference, *P* = 0.002, respectively) (Fig. 6A).

It is known that ERK/MAPK pathway plays a major role in cell proliferation and survival. Therefore, we performed a Western blot analysis to assess ERK activation, by using antibodies either specific for the phosphorylated form of the molecule or directed against total ERK. As expected, in mock-transfected cells ERK activation was significantly induced by KGF treatment (7.5 fold). Conversely, 5-FU was able to decrease the levels of phosphorylated ERK (2.5 fold), which were almost completely restored by the administration of KGF together with 5-FU (6.5 fold). In siKGFR-transfected cells, according to the above results, the stimulating effect of KGF on ERK phosphorylation was greatly inhibited (2 fold) (Fig. 6B).

**Figure 7:**
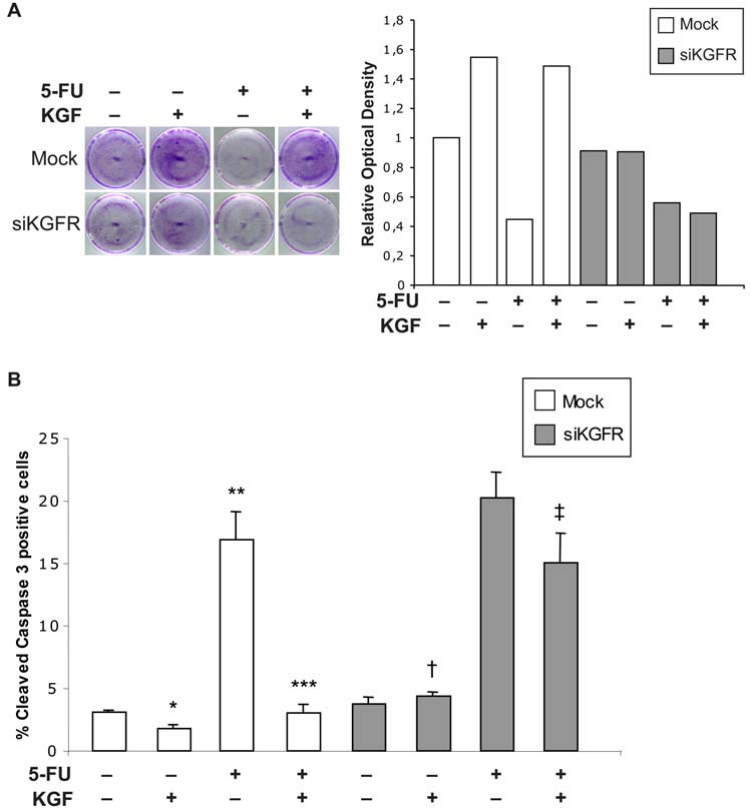
Effect of siKGFR transfection on cell viability and KGF-induced inhibition of 5-FU proapoptotic activity. (A) MCF-7 cells were mock transfected or transfected with 5 nM siKGFR and 48 h later they were treated or not with 20 ng/ml KGF, 25 µg/ml 5-FU or KGF plus 5-FU. After 24 h, cells were fixed, stained with 1% crystal violet and analyzed at an absorbance of 570 nm. The values from a representative experiment were expressed as relative optical density and reported as a graph. (B) MCF-7 cells, grown on coverslips, were transfected and treated as in (A). After 24 h, they were fixed and subjected to immunofluorescence analysis with an antibody directed against the cleaved form of caspase-3. Nuclei were visualized using 4′, 6-diamido-2-phenylindole dihydrochloride (DAPI). The percentage of apoptotic cells was evaluated by counting the number of cleaved caspase-3 positive nuclei versus total number of nuclei in ten different areas randomly taken from three different experiments, expressed as mean value±95% CI and reported as a graph. *P* values were determined using the Student's *t* test: * *P* = 0.001 and ** *P* = 0.001 versus untreated mock-transfected cells; *** *P*<0.001 versus 5-FU-treated mock-transfected cells; † *P*<0.001 versus KGF-treated mock-transfected cells; ‡ *P* = 0.001 versus KGF plus 5-FU-treated mock-transfected cells.

Furthermore, we assessed cell viability by crystal violet staining, and its subsequent absorbance at 570 nm, which reflects variations in cell number. 48 h after transfection, cells were treated with KGF, 5-FU or a combination of them for 24 h, as described above. Then, the remaining cells were fixed and stained with 1% crystal violet (Fig. 7A). We compared the effect of KGF and 5-FU on cell number, looking at the influence of KGFR silencing in this process. In mock-transfected cells KGF treatment was able to increase cell number (1.55 fold), 5-FU induced a decrease of viable cells (0.45 fold), whereas in presence of KGF, 5-FU was not effective (1.49 fold). On the other hand, in siKGFR-transfected cells, 5-FU induced cell death as expected (0.56 fold), whereas treatment with KGF was not able to induce an increase of cell number (0.91 fold). In this case 5-FU keeps its ability to determine cell death even in presence of KGF (0.49 fold) (Graph Fig. 7A).

In conclusion, these results highlight the ability of siKGFR to counteract the capacity of KGF to block the antiproliferative effect of chemotherapeutic agents, such as 5-FU.

### KGFR silencing abrogates the antiapoptotic effects of KGF upon 5-FU stimulation

Since cell treatment with 5-FU is known to induce apoptosis, we assayed the protective role of KGF towards 5-FU induction of apoptosis in MCF-7 cells [21], [22]. MCF-7 cells were transfected with siKGFR or mock-transfected and grown for 48 h in standard medium supplemented or not with KGF, 5-FU or a combination of them, as above. Cell apoptosis was evaluated by counting cells positive for the cleaved, active form of caspase-3, a key executioner of apoptosis, and reported in graph as percentage of positive cells (Fig. 7B). KGF was able to induce a slight reduction in cell apoptosis with respect to untreated cells (1.8% versus 3.1%, 1.7 fold difference, *P* = 0.001), while the treatment with 5-FU caused a strong apoptotic effect (16.9% versus 3.1%, 5.5 fold increase, *P* = 0.001). As previously demonstrated on the same cellular model [21], the combination with KGF is able to protect MCF-7 cells from apoptosis induced by 5-FU (3.0% versus 16.9%, 5.6 fold difference, *P*<0.001). KGFR silencing suppressed KGF antiapoptotic effect, both alone and in combination with 5-FU (4.4% versus 1.8%, 2.4 fold difference, *P*<0.001 and 15.1% versus 3.0%, 5.0 fold difference, *P* = 0.001, respectively) (Fig. 7B).

These data were consistent with the results obtained by Q-RT-PCR, Western blot analysis and proliferation assay, and showed that knockdown of KGFR protein expression may be a therapeutic approach to avoid KGF suppression of 5-FU-induced apoptosis in cancer cells.

### KGFR silencing restores the antiproliferative effect of tamoxifen on ER positive cells

**Figure 8:**
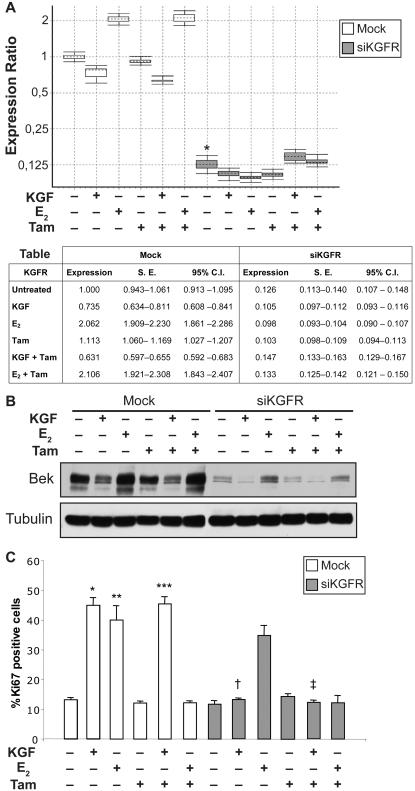
Effect of siKGFR transfection on KGF-induced inhibition of Tamoxifen antiproliferative activity in MCF-7 cells. (A) MCF-7 cells were mock transfected or transfected with 5 nM siKGFR. 48 h after transfection, cells were treated with 20 ng/ml KGF, 20 ng/ml E_2_, 100 nM tamoxifen (Tam), KGF plus Tam or E_2_ plus Tam for 24 h. The levels of KGFR mRNA expression were determined by Q-RT-PCR, and normalized to β-actin mRNA levels. The amount of KGFR mRNA in siKGFR-transfected cells was expressed as fold of the level of KGFR mRNA with respect to untreated mock-transfected cells. For each treatment, the graph shows the interquartile range of three independent experiments (boxes), their mean (horizontal dotted bars), and 95% CI (whiskers). The accompanying table reports mean expression level, Standard Error (S.E.), and 95% CI for each assayed sample. *P* values were determined using the Student's *t* test: * *P*<0.001 versus untreated mock-transfected cells. (B) MCF-7 cells were transfected and treated as in (A), and the amount of KGFR protein was evaluated by Western blot analysis with an anti-bek polyclonal antibody. Tubulin served as a loading control. (C) MCF-7 cells, grown on coverslips, were transfected and treated as in (A). After 24 h, cells were fixed and subjected to immunofluorescence analysis with an anti-Ki67 polyclonal antibody. Nuclei were visualized using 4′, 6-diamido-2-phenylindole dihydrochloride (DAPI). Cell proliferation was evaluated as percentage of Ki67-positive nuclei versus total number of nuclei in ten different areas randomly taken from three different experiments, expressed as mean value±95% CI and reported as a graph. *P* values were determined using the Student's *t* test: * *P*<0.001 and ** *P* = 0.001 versus untreated mock-transfected cells; *** *P*<0.001 versus Tam-treated mock-transfected cells; † *P*<0.001 versus KGF-treated mock-transfected cells; ‡ *P*<0.001 versus KGF plus Tam-treated mock-transfected cells.

Tamoxifen is the most frequently prescribed anti-estrogen for the management of estrogen-responsive human breast cancers. Nevertheless, many tamoxifen responsive breast cancer patients acquire tamoxifen resistance, which mechanisms are not completely understood [26]. A potential interaction with the FGF/FGFR pathways has been hypothesized to be involved in this process, although not yet clarified [27]. To assess the possible contribution of KGF/KGFR to the establishment of tamoxifen resistance, we analyzed the effects of KGF, 17β-estradiol (E_2_) and tamoxifen treatment on MCF-7 cells. The transfection with siKGFR was performed in the presence or not of 20 ng/ml KGF, 20 ng/ml E_2_, 100 nM tamoxifen or combinations of them. As shown in Figure 8A–B, at RNA and protein level, respectively, in mock-transfected cells KGF induced a decrease of KGFR expression both with (0.631 fold, reduction = 36.9%, *P*<0.001) and without (0.735 fold, reduction = 26.5%, *P* = 0.049) co-treatment with tamoxifen. Tamoxifen alone slightly affected KGFR mRNA expression (1.113 fold, difference = 11.3%, *P* = 0.032), while E_2_ caused an increase of more than two fold of KGFR expression (2.062 fold, difference = 106.2%, *P*<0.001), which was not affected by co-treatment with tamoxifen (2.106 fold, difference = 110.6%, *P*<0.001). On the other hand, in siKGFR-transfected cells a strong reduction of KGFR expression was observed (0.126 fold, reduction = 87.4%, *P*<0.001), and it was not affected by treament with KGF, tamoxifen and E_2_ alone or in combination.

The same cultures were subsequently assayed to determine proliferation rates by counting cells positive for Ki67 antigen. Data were reported in graph as percentage of positive cells (Fig. 8C). In mock-transfected cells, we observed an increase in cell proliferation after KGF treatment, as compared to untreated cells (45% versus 13.2%, 3.4 fold increase, *P*<0.001). As previously reported [35], [36], also E_2_ caused an induction of MCF-7 cells proliferation (40.1% versus 13.2%, 3.0 fold increase, *P* = 0.001). Tamoxifen treatment induced only a slight reduction of basal proliferation rate (12.1% versus 13.2%, 0.9 fold difference, *P* = 0.065) that was not altered by co-treatment with E_2_ (12.2% versus 12.1%, *P* = 0.787), while its effect was efficiently counteracted by KGF (45.5% versus 12.1%, 3.8 fold increase, *P*<0.001). KGFR silencing did not significantly affect E_2_-induced cell proliferation (34.8% versus 40.1%, 1.2 fold difference, *P* = 0.129), whereas in silenced cells KGF was not able to stimulate cell proliferation both in absence and presence of tamoxifen (13.2% versus 45%, 3.4 fold difference, *P*<0.001 and 12.3% versus 45.5%, 3.7 fold difference, *P*<0.001, respectively).

**Figure 9:**
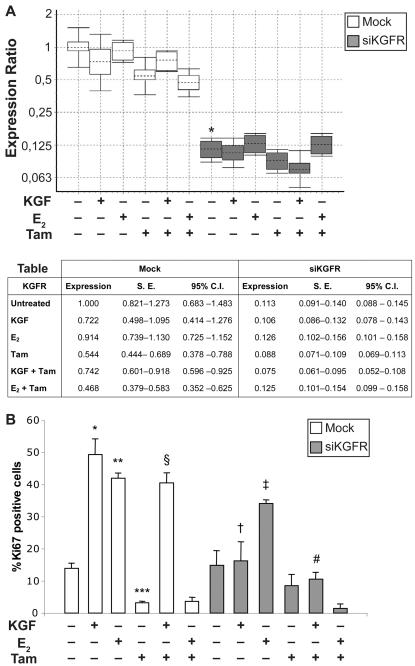
Effect of siKGFR transfection on KGF-induced inhibition of Tamoxifen antiproliferative activity in KCs cells. (A) KCs cells were mock transfected or transfected with 5 nM siKGFR. 48 h after transfection, cells were treated with 20 ng/ml KGF, 20 ng/ml E_2_, 100 nM Tam, KGF plus Tam or E_2_ plus Tam for 24 h. The levels of KGFR mRNA expression were determined by Q-RT-PCR, and normalized to β-actin mRNA levels. The amount of KGFR mRNA in siKGFR-transfected cells was expressed as fold of the level of KGFR mRNA with respect to untreated mock-transfected cells. For each treatment, the graph shows the interquartile range of three independent experiments (boxes), their mean (horizontal dotted bars), and 95% CI (whiskers). The accompanying table reports mean expression level, Standard Error (S.E.), and 95% CI for each assayed sample. *P* values were determined using the Student's *t* test: * *P* = 0.002 versus untreated mock-transfected cells. (B) KCs cells, grown on coverslips, were transfected and treated as in (A). After 24 h, cells were fixed and subjected to immunofluorescence analysis with an anti-Ki67 polyclonal antibody. Nuclei were visualized using 4′, 6-diamido-2-phenylindole dihydrochloride (DAPI). Cell proliferation was evaluated as percentage of Ki67-positive nuclei versus total number of nuclei in ten different areas randomly taken from three different experiments, expressed as mean value±95% CI and reported as a graph. *P* values were determined using the Student's *t* test: * *P*<0.001, ** *P*<0.001 and *** *P*<0.001 versus untreated mock-transfected cells; § *P*<0.001 versus Tam-treated mock-transfected cells; † *P*<0.001 versus KGF-treated mock-transfected cells; ‡ *P*<0.001 versus E_2_- treated mock-transfected cells; # *P*<0.001 versus KGF plus Tam-treated mock-transfected cells.

The same set of experiments was then performed on primary cultures of KCs (Fig. 9). Treatment with KGF, alone or in combination with tamoxifen, barely reduced the expression of KGFR mRNA compared to untreated cells, (0.722 fold, reduction = 27.8%, *P* = 0.283, and 0.742 fold, reduction = 25.8%, *P* = 0.134, respectively) and a decrease of KGFR mRNA expression (0.544 fold, difference = 45.6%, *P* = 0.020) was also observed in tamoxifen treated cells. Transfection with siKGFR induced a significant decrease in KGFR expression (0.113 fold, reduction = 88.7%, *P* = 0.002), with negligible variations due to the presence of KGF, E_2_ and/or tamoxifen (Fig. 9A). As for the proliferation rates, reported in Figure 9B, a strong increase was observed in KGF as well as E_2_ treated cells (49.3% versus 13.9%, 3.5 fold increase, *P*<0.001 and 41.9% versus 13.9%, 3.0 fold increase, *P*<0.001, respectively). Tamoxifen caused a decrease in cell proliferation as compared to untreated cells (3.2% versus 13.9%, 4.3 fold difference, *P*<0.001) even in presence of E_2_ (3.6% versus 3.2%, 1.1 fold difference, *P* = 0.756). However, tamoxifen effect was abolished by KGF-induced cell growth in co-treated cultures (40.5% versus 3.2%, 12.6 fold increase, *P*<0.001). In KGFR silenced KCs, a strong reduction of the capacity of KGF to induce cell proliferation was observed (16.2% versus 15%, 1.1 fold increase, *P* = 0.762). Even in this case, E_2_ capacity is only partially affected (34.1% versus 41.9%, 1.2 fold difference, *P*<0.001). However, in silenced cells tamoxifen blockade of cell proliferation was not counteracted by KGF (10.5% versus 40.5%, 3.9 fold difference, *P*<0.001)

Taken altogether, these results highlight the ability of siKGFR to prevent KGF from blocking the antiproliferative effect of tamoxifen in ER positive cells.

## Discussion

In the present study we took advantage of the mechanism of RNAi to silence the expression of KGFR in epithelial cell lines. Thus, we analyzed the effect of siKGFR on cell proliferation and motility in transiently transfected cells. We also evaluated whether downregulation of KGFR expression could restore the apoptotic and antiproliferative effects of 5-FU and tamoxifen on a breast cancer cell line as well as on epithelial primary cultures.

Alterations in the expression of growth factors and/or their high affinity receptors have been shown to be involved in processes that can lead to tumor development. The FGFR family and the related ligands participate in the physiological processes that regulate differentiation, proliferation, migration and cell survival [37]. Many splice variants generated from the 4 genes encoding the FGFRs and 23 FGF ligands thus far identified yield a high number of combinations ensuring a finely regulated cross-talk between epithelial and mesenchymal cells. Moreover, proper FGFR signaling requires restricted expression of specific receptors on different cell types. Among FGFR, KGFR expression turned out to be tissue specific, being expressed on epithelial cells under physiological conditions [2].

Previous studies have reported that abnormal expression of KGFR can be correlated with tumor progression, reduction of free survival and worse prognosis in different epithelial cancers, such as prostate, breast, gastric [38] and pancreatic [39] carcinomas. In particular, in prostate cancer, lack of KGFR expression seems to be associated to a more aggressive behavior of the tumor, which becomes androgen-insensitive [40]. In salivary gland tumors, loss of KGFR expression has been reported in malignant transformation [41]. On the other hand, in pancreatic cancer it has been observed that co-expression of KGF and KGFR in tumor cells is correlated with poorer prognosis. It has been hypothesized that upregulation of either KGF or KGFR expression may contribute to venous invasion, possibly through induction of VEGF-A expression, thus causing a higher risk of metastasis [39]. In breast cancer, KGFR upregulation has been documented in specimens obtained at the early stages of tumors [42]. Thus, it has been suggested that KGF-mediated stimulation of these cells might contribute to the metastatic progression. KGF has been also observed in some tumor cells of epithelial origin, despite its physiologic expression is restricted to mesenchymal cells. This finding has been documented in about a half of breast cancers and a quarter of pancreatic adenocarcinomas, suggesting a potential autocrine loop of the KGF/KGFR axis in these tumor cells [21], [43], [44]. Co-expression of KGF and KGFR has been also documented in the MCF-7 cell line. It has been suggested that co-expression of KGF/KGFR in tumor cells might interfere with the efficacy of some chemotherapeutic agents, thus contributing to a poorer prognosis of the disease [21]. Furthermore, it has been observed that KGF, following the binding to KGFR, exerts an antiapoptotic role in epithelial cells, by affecting the regulation of AKT/MAPK survival/proliferation pathway, whose end results influence cell fate [45].

Moreover, acquired resistance to tamoxifen therapy in ER-positive breast cancers is a frequent clinical observation, although the mechanisms capable to determine this effect remain unclear. Deregulations involving members of the FGF/FGFR family have been suggested to play a role in this phenomenon [27].

In the present study we set up a system that efficiently reduces KGFR expression in epithelial cells. At our knowledge, this is the first highly specific RNAi active on KGFR without affecting the closely related FGFR2-IIIc isoform. This finding might turn out important to evaluate the effects on epithelial tumors characterized by an altered activation of KGFR. Furthermore, it could represent a useful approach to selectively study the pathway of KGFR activation without interfering with other FGFR family members.

We observed that silencing of KGFR caused modifications in the physiological behavior of the tested cell lines. Two major effects of KGF treatment on epithelial cells were assayed. Both proliferation rate, as measured by Ki67 marker, and migration capacity, as determined by the *in vitro* wound-healing assay, showed that KGFR silenced cells become poorly responsive to KGF. These modifications of cell behavior are particularly intriguing since high rates of proliferation and migration represent two fundamental characteristics of malignant cells. It could be envisioned the possibility that KGFR silencing in cancers, particularly in those overexpressing KGFR and/or KGF, might affect the growth rate of the primary tumor as well as its metastatic potential.

A further interesting observation was obtained by evaluating the effects of 5-FU in KGFR silenced cells. Previous researches have shown that KGF interferes with the capacity of 5-FU to block cell proliferation and to induce apoptosis [19], [21]. We confirmed these data in our study, but more importantly we observed that silencing of KGFR expression restores the efficacy of 5-FU, as documented by Ki67 labeling, index of cell proliferation, and by the viability assay, measure of the capacity of 5-FU to cause cell death. These results underline the potential role of the KGF/KGFR pathway in decreasing the therapeutic efficacy of 5-FU treatment.

Similarly, in the breast cancer cell line MCF-7 and in epithelial primary cultured cells, we observed that KGF treatment interferes with the capacity of tamoxifen to block cell proliferation, whereas KGFR silencing completely restores tamoxifen efficacy.

Although not clearly established, the involvement of the KGF/KGFR pathway has been envisioned in different tumors of epithelial origin. Recent data showing a possible association between genetic alterations in the FGFR2 gene and risk of breast cancers raised the interest to studies aimed to better address this issue [46], [47]. The altered expression of KGF/KGFR observed in some cancers suggests that a screening to evaluate the expression of KGFR and KGF in tumor biopsies might turn out useful as prognostic marker. The present study, showing that silencing of KGFR affects proliferation, motility and response of tumor-derived cell lines to chemotherapeutic drugs, such as 5-FU and tamoxifen, seems to indicate that KGFR may represent an important target for the development of novel therapeutic strategies.

Further studies are needed to prove specificity and efficacy of siKGFR *in vivo*. Should these studies confirm our findings, it could be envisioned the possibility that delivery of siRNAs within tumor cells to downregulate KGFR expression might represent an approach to overcome the reduced efficacy of drugs commonly used in the treatment of tumors of epithelial origin, as well as to reduce the rate of tumor growth and metastasis.

## Materials and Methods

### Cell culture and treatments

The estrogen receptor α-positive MCF-7 human breast adenocarcinoma cell line, purchased from the American Type Culture Collection (No. HTB-22, ATCC-LGC Promochem, Teddington, UK), and the human keratinocyte cell line HaCaT were both cultured in Dulbecco's Modified Eagle's Medium (DMEM; Invitrogen, Karlsruhe, Germany), supplemented with 10% fetal bovine serum (FBS; Invitrogen) and antibiotics. Primary cultures of human estrogen-sensitive keratinocytes (KCs) were established from 1 cm^2^ full-thickness mucosal biopsy of the vaginal vestibule. Following enzymatic dissociation, keratinocytes were seeded onto collagen IV (10 mg/ml)-coated culture plates and maintained in chemical defined medium MCDB 153 (EpiLife, Cascade Biologics, Inc., Portland, OR, USA), with medium change twice a week, as previously reported [48].

For quantitative RT-PCR (Q-RT-PCR), Western blot analysis and immunofluorescence, 20 h after transfection with siRNA HaCaT cells were serum starved for 4 h and then treated for 48 h with 20 ng/ml human recombinant KGF (Upstate Biotechnology, Lake Placid, NY), while MCF-7 cells were treated with 20 ng/ml KGF, 25 µg/ml 5-FU (Sigma-Aldrich, Milan, Italy) or a combination of them. For wound-healing assay, 36 h after transfection cells were serum starved for 12 h and then treated for 24 h with 50 ng/ml KGF. For the experiments with 17β-estradiol (E_2_) and tamoxifen, MCF-7 cells were grown in phenol red-free DMEM supplemented with 10% dextran charcoal-treated FBS (Invitrogen). Both MCF-7 and KCs were serum starved for 4 h and then treated for 48 h with 20 ng/ml KGF, 20 ng/ml E_2_ (Sigma-Aldrich), 100 nM tamoxifen or combinations of them.

### Design and selection of siRNAs

The targeted sequences for human KGFR siRNAs, selected from the cDNA sequence located <0.2 kb after start codon, were designed by using publicly available algorithms (www.ambion.com/techlib/misc/siRNA_finder.html) and according to the guidelines from Tuschl et al. [49]. In brief, we selected three 27-mer RNA duplexes targeted to sequences located within the exon 8 of the FGFR2 gene, exclusively expressed in the KGFR/FGFR2-IIIb isoform, and not in the FGFR2-IIIc. These duplexes, named siRNA-1, siRNA-2 and siRNA-3, were analyzed by BLAST (http://www.ncbi.nlm.nih.gov/BLAST) to ensure that there were no significant sequence homologies with other genes. Subsequently, they were synthesized by Invitrogen and dissolved in the siRNAs buffer, as recommended by the manufacturer. The efficacy of either the three individual duplexes or the pooled set of them was assessed by Q-RT-PCR, the most efficient condition was chosen for following studies and it was referred to as siKGFR.

### *In vitro* transfection with siRNAs

HaCaT, MCF-7 and KCs cells were transfected with siRNAs using the HiPerfect transfection reagent (Qiagen Inc., Hilden, Germany), according to the manufacturer's instructions. Briefly, 1 day prior to transfection cells were seeded at 1.5×10^5^ for HaCaT or 2.0×10^5^ for MCF-7 and KCs per 60 mm Petri dish, corresponding to a density of 60%-70% at the time of transfection. The final optimal siRNA concentration was determined in 5 nM (data not shown). Cells were incubated with HiPerfect alone without siRNA as a negative control (mock transfection). Cells were harvested 72 h after transfection for mRNA analysis, protein expression, immunofluorescence and wound-healing assays. In time course experiments, the analysis was performed 6, 24, 48, 72 and 96 h after transfection. In each case, three replicate experiments were performed.

### Quantitative RT-PCR

Cells were harvested and total RNA was extracted with the use of TRIzol reagent (Invitrogen). cDNA was generated with oligo(dT) from 1 µg of RNA using the SuperScript III Reverse Transcriptase Kit (Invitrogen). After reverse transcription, the abundance of KGFR or FGFR2-IIIc mRNA in HaCaT and MCF-7 cells was quantified by Q-RT-PCR. Relative quantification was performed using β-actin mRNA as an endogenous control: for each examined sample, KGFR and FGFR2-IIIc mRNA expression data were normalized to the β-actin expression. The primers sets (Invitrogen) designed to detect each mRNA were the following: KGFR forward, 5′-ACTCGGGGATAAATAGTTCCAA-3′; KGFR reverse, 5′-CCTTACATATATATTCCCCAGCAT-3′; FGFR2-IIIc forward, 5′-CACCACGGACAAAGAGATTGA-3′; FGFR2-IIIc reverse, 5′- ATTACCCGCCAAGCACGTAT-3′; β-actin forward, 5′- CGCCGCCAGCTCACCATG-3′; β-actin reverse, 5′-CACGATGGAGGGGAAGACGG-3′. *Taq*Man probes for KGFR (5′-AAGTGCTGGCTCTGTTCAATGT-3′), FGFR2-IIIc (5′-TGTAACTTTTGAGGACGCTGGGGAA-3′) and β-actin (5′-TCGACAACGGCTCCGGCATGTGCA-3′) were purchased from MWG-BIOTECH AG (Anzingerstr, Ebersberg, Germany). The Q-RT-PCR reactions were performed using iQ Supermix (Bio-Rad Laboratories, Hercules, CA) in a MyiQ™ Thermal Cycle (Bio-Rad), following the manufacturer's instructions. For each sample three replicates were performed. All reactions began with 3 min at 95°C for iTaq™ DNA polymerase activation, followed by 40 cycles of 95°C for 15 sec for denaturation and 60°C for 1 min for annealing-extension. Data were analyzed according to Pfaffl [50] and were expressed as fold of KGFR or FGFR2-IIIc mRNA with respect to control. For each graph, boxes indicated the interquartile range, the horizontal dotted line indicated the mean value and whiskers indicated 95% confidence intervals (CIs).

### Western blot analysis

For Western blot analysis, siKGFR- and mock-transfected HaCaT or MCF-7 cells, treated as above described, were lysed in RIPA buffer. Total proteins (50–150 µg) were resolved under reducing conditions by 8%–12% SDS–PAGE and transferred to Immobilon-FL membranes (Millipore, Billerica, MA). For KGFR detection, the membranes were incubated overnight at 4°C with anti-bek, a rabbit polyclonal antibody raised against the intracellular domain of KGFR/FGFR2 (C-17; 1∶200 dilution; Santa Cruz Biotechnology, Santa Cruz, CA), followed by a goat anti-rabbit horseradish peroxidase-conjugated secondary antibody (Sigma-Aldrich). Bound antibody was detected by enhanced chemiluminescence detection reagents (Pierce Biotechnology, Inc, Rockford, IL), according to manufacturer's instructions. To estimate the protein equal loading, the membranes were rehydrated through washing in TBS–T, stripped with 100 mM β-mercaptoethanol and 2% SDS for 30 min at 55°C and reprobed with an anti-tubulin antibody (1∶1000 dilution; Sigma-Aldrich). To detect MAPK activation, membranes were incubated overnight at 4°C with a monoclonal antibody that recognizes the phosphorylated form of ERK (1∶1000 dilution; Cell Signaling Technology, Danvers, MA), followed by goat anti-mouse horseradish peroxidase-conjugated secondary antibody (Sigma-Aldrich), which was visualized by enhanced chemiluminescence. Protein equal loading was assessed by reprobing the membranes with a polyclonal antibody directed against ERK-2 (1∶1000 dilution; Sigma-Aldrich). Densitometric analysis was performed using Quantity One Program (Bio-Rad). Briefly, the signal intensity for each band was calculated and the background subtracted from experimental values. The resulting values were then normalized, expressed as fold increase with respect to the control value and visualized as graphs.

### Immunofluorescence microscopy

Cells, grown on coverslips, were siKGFR- or mock-transfected and treated as described above, then fixed in 4% paraformaldehyde in phosphate-buffered saline (PBS) for 30 min at 25°C, followed by treatment with 0.1 M glycine in PBS for 20 min at 25°C and with 0.1% Triton X-100 in PBS for additional 5 min at 25°C to allow permeabilization. To assess cell proliferation, cells were incubated with an anti-Ki67 rabbit polyclonal antibody (1∶50 in PBS; Zymed Laboratories, San Francisco, CA), which identifies cycling cells. The primary antibody was visualized using Texas Red conjugated goat anti-rabbit IgG (1∶100 in PBS; Jackson ImmunoResearch Laboratories, West Grove, PA). To evaluate apoptosis, cells were incubated with a primary antibody that specifically detects the cleaved form of caspase-3 (1∶400 in PBS; Cell Signaling), visualized with a FITC-conjugated goat anti-rabbit IgG (1∶50 in PBS; Cappel Research Products, Durham, NC). Nuclei were visualized using 4′, 6-diamido-2-phenylindole dihydrochloride (DAPI) (1∶10000 in PBS; Sigma-Aldrich). Fluorescence signals were analyzed by recording stained images using a cooled CCD color digital camera SPOT-2 (Diagnostic Instruments Incorporated, Sterling Heights, MI) and Axiovision software (Carl Zeiss Inc., Oberkochen, Germany). The percentage of Ki67-positive cells and of cleaved-caspase 3-positive cells was evaluated by counting, for each treatment, a total of 500 cells, randomly taken from ten microscopic fields in three different experiments, expressed as mean value±95% CI and reported as graphs.

### Cell survival assay

To evaluate the cytotoxicity of 5-FU, siKGFR- or mock-transfected MCF-7 cells were treated as described above, fixed for 10 min in a solution 10% acetic acid-10% methanol, stained with crystal violet (1% w/v) and photographed using a Power Shot G5 digital camera (Canon, Inc., Tokyo, Japan). Since it is known that the intensity of light passing through the crystal violet stained culture is proportional to the number of cells per unit area [51], we further measured the optical density of the stained cells at a wavelength of 570 nm, and reported it as a graph.

### Wound healing assay

HaCaT and MCF-7 cells were seeded at 2×10^5^ cells and 1.2×10^5^ cells per 35 mm Petri dish, respectively, transfected as described above and grown until confluence. Confluent cells were serum starved for 12 h and then a standardized cell-free area (wound) was introduced by scraping the monolayer with a sterile tip, as previously described [33]. After intensive wash, the remaining cells were incubated for 24 h in the presence of 50 ng/ml KGF. Then, cells were fixed with 4% paraformaldehyde for 30 min at 25°C and photographs were taken using an Axiovert 25 inverted microscope (Carl Zeiss) and a Power Shot G5 digital camera (Canon, Inc.). Some plates were fixed and photographed immediately after wounding, representing a T0 control. Migration was quantified by a measure of the recovered wound area, performed using the freely available image-processing software ImageJ 1.38 (http://rsb.info.nih.gov/ij/). The data presented for each cell line are a mean of triplicate experiments±95% CI.

### Statistical analysis

Data were analyzed by two-way analysis of variance (ANOVA) and Student's *t* test. 95% confidence intervals were calculated. All statistical tests were two-sided, and *P*<0.05 were considered statistically significant. Statistical analyses were performed using STATA 8.0 (STATA Corporation, College Station, TX).

## References

[1] Miki T, Bottaro DP, Fleming TP, Smith CL, Burgess WH (1992). Determination of ligand binding specificity by alternative splicing: two distinct growth factor receptors encoded by a single gene.. Proc Natl Acad Sci U S A.

[2] Orr-Urtreger A, Bedford MT, Burakova T, Arman E, Zimmer Y (1993). Developmental localization of the splicing alternatives of fibroblast growth factor receptor-2 (FGFR2).. Dev Biol.

[3] Finch PW, Rubin JS, Miki T, Ron D, Aaronson SA (1989). Human KGF is FGF-related with properties of a paracrine effector of epithelial cell growth.. Science.

[4] Eswarakumar VP, Lax I, Schlessinger J (2005). Cellular signaling by fibroblast growth factor receptors.. Cytokine Growth Factor Rev.

[5] Marchese C, Rubin JS, Ron D, Faggioni A, Torrisi MR (1990). Human keratinocyte growth factor activity on proliferation and differentiation of human keratinocytes: differentiation response distinguishes KGF from EGF family.. J Cell Physiol.

[6] Hines MD, Allen-Hoffman BL (1996). Keratinocyte growth factor inhibits cross-linked envelope formation and nucleosomal fragmentation in cultured human keratinocytes.. J Biol Chem.

[7] Lu Y, Pan ZZ, Devaux Y, Ray P (2003). p21-activated protein kinase 4 (PAK 4) interacts with the keratinocyte growth factor receptor and participates in keratinocyte growth factor-mediated inhibition of oxidant-induced cell death.. J Biol Chem.

[8] Staiano-Coico L, Krueger JG, Rubin JS, D'limi S, Vallat VP (1993). Human keratinocyte growth factor effects in a porcin model of epidermal wound healing.. J Exp Med.

[9] Werner S, Smola H, Liao X, Longaker MT, Krieg T (1994). The function of KGF in morphogenesis of epithelium and reepithelialization of wounds.. Science.

[10] Marchese C, Chedid M, Dirsch OR, Csaky KG, Santanelli F (1995). Modulation of keratinocyte growth factor and its receptor in reepithelializing human skin.. J Exp Med.

[11] Tsuboi R, Sato C, Kurita Y, Ron D, Rubin JS (1993). Keratinocyte growth factor (FGF-7) stimulates migration and plasminogen activator activity of normal human keratinocytes.. J Invest Dermatol.

[12] Sato C, Tsuboi R, Shi CM, Rubin JS, Ogawa H (1995). Comparative study of hepatocyte growth factor/scatter factor and keratinocyte growth factor effects on human keratinocytes.. J Invest Dermatol.

[13] Galiacy S, Planus E, Lepetit H, Fereol S, Laurent V (2003). Keratinocyte growth factor promotes cell motility during alveolar epithelial repair in vitro.. Exp Cell Res.

[14] Finch PW, Rubin JS (2006). Keratinocyte growth factor expression and activity in cancer: implications for use in patients with solid tumors.. J Natl Cancer Inst.

[15] Zang XP, Pento JT (2000). Keratinocyte growth factor-induced motility of breast cancer cells.. Clin Exp Metastasis.

[16] Nguyen TN, Zang XP, Pento JT (2002). Keratinocyte growth factor stimulates the migration and proliferation of breast cancer cells in a culture wounding model.. Pharmacol Res.

[17] Zang XP, Lerner MR, Dunn ST, Brackett DJ, Pento JT (2003). Antisense KGFR oligonucleotide inhibition of KGF-induced motility in breast cancer cells.. Anticancer Res.

[18] Shin EY, Lee BH, Yang JH, Shin KS, Lee GK (2000). Up-regulation and co-expression of fibroblast growth factor receptors in human gastric cancer.. J Cancer Res Clin Oncol.

[19] Farrell CL, Bready JV, Rex KL, Chen JN, DiPalma CR (1998). Keratinocyte growth factor protects mice from chemotherapy and radiation-induced gastrointestinal injury and mortality.. Cancer Res.

[20] Crescioli C, Maggi M, Luconi M, Vannelli GB, Salerno R (2002). Vitamin D3 analogue inhibits keratinocyte growth factor signaling and induces apoptosis in human prostate cancer cells.. Prostate.

[21] Tamaru N, Hishikawa Y, Ejima K, Nagasue N, Inoue S (2004). Estrogen receptor-associated expression of keratinocyte growth factor and its possible role in the inhibition of apoptosis in human breast cancer.. Lab Invest.

[22] Hishikawa Y, Tamaru N, Ejima K, Hayashi T, Koji T (2004). Expression of keratinocyte growth factor and its receptor in human breast cancer: its inhibitory role in the induction of apoptosis possibly through the overexpression of Bcl-2.. Arch Histol Cytol.

[23] Tsukioka Y, Matsumura Y, Hamaguchi T, Goto M, Muro K (2001). Complete response achieved following administration of S-1 in a patient with adrenal gland metastasis of 5-FUresistant gastric cancer: a case report.. Jpn J Clin Oncol.

[24] Zhang L, Kharbanda S, Hanfelt J, Kern FG (1998). Both autocrine and paracrine effects of transfected acidic fibroblast growth factor are involved in the estrogen-independent and antiestrogen-resistant growth of MCF-7 breast cancer cells.. Cancer Res.

[25] McLeskey SW, Zhang L, El-Ashry D, Trock BJ, Lopez CA (1998). Tamoxifen-resistant fibroblast growth factor-transfected MCF-7 cells are cross-resistant in vivo to the antiestrogen ICI 182,780 and two aromatase inhibitors.. Clin Cancer Res.

[26] Chang HL, Sugimoto Y, Liu S, Ye W, Wang LS (2006). Keratinocyte growth factor (KGF) induces tamoxifen (Tam) resistance in human breast cancer MCF-7 cells.. Anticancer Res.

[27] Adamo V, Iorfida M, Montalto E, Festa V, Garipoli C (2007). Overview and new strategies in metastatic breast cancer (MBC) for treatment of tamoxifen-resistant patients.. Ann Oncol.

[28] Tschaharganeh D, Ehemann V, Nussbaum T, Schirmacher P, Breuhahn K (2007). Non-specific effects of siRNAs on tumor cells with implications on therapeutic applicability using RNA interference.. Pathol Oncol Res.

[29] Kim DH, Behlke MA, Rose SD, Chang MS, Choi S (2005). Synthetic dsRNA Dicer substrates enhance RNAi potency and efficacy.. Nat Biotechnol.

[30] Nagy N, Bata-Csörgo Z, Kopasz N, Szeg C, Pivarcsi A (2006). The expression of keratinocyte growth factor receptor (FGFR2-IIIb) correlates with the high proliferative rate of HaCaT keratinocytes.. Exp Dermatol.

[31] Nurcombe V, Smart CE, Chipperfield H, Cool SM, Boilly B (2000). The proliferative and migratory activities of breast cancer cells can be differentially regulated by heparan sulfates.. J Biol Chem.

[32] Grimm D, Streetz KL, Jopling CL, Storm TA, Pandey K (2006). Fatality in mice due to oversaturation of cellular microRNA/short hairpin RNA pathways.. Nature.

[33] Cha D, O'Brien P, O'Toole EA, Woodley DT, Hudson LG (1996). Enhanced modulation of keratinocyte motility by transforming growth factor-alpha (TGF-alpha) relative to epidermal growth factor (EGF).. J Invest Dermatol.

[34] Ceccarelli S, Cardinali G, Aspite N, Picardo M, Marchese C (2007). Cortactin involvement in the keratinocyte growth factor and fibroblast growth factor 10 promotion of migration and cortical actin assembly in human keratinocytes.. Exp Cell Res.

[35] Huseby RA, Maloney TM, McGrath CM (1984). Evidence for a direct growth-stimulating effect of estradiol on human MCF-7 cells in vivo.. Cancer Res.

[36] Lee YR, Park J, Yu HN, Kim JS, Youn HJ (2005). Up-regulation of PI3K/Akt signaling by 17beta-estradiol through activation of estrogen receptor-alpha, but not estrogen receptor-beta, and stimulates cell growth in breast cancer cells.. Biochem Biophys Res Commun.

[37] Grose R, Dickson C (2005). Fibroblast growth factor signaling in tumorigenesis.. Cytokine Growth Factor Rev.

[38] Matsunobu T, Ishiwata T, Yoshino M, Watanabe M, Kudo M (2006). Expression of keratinocyte growth factor receptor correlates with expansive growth and early stage of gastric cancer.. Int J Oncol.

[39] Cho K, Ishiwata T, Uchida E, Nakazawa N, Korc M (2007). Enhanced expression of keratinocyte growth factor and its receptor correlates with venous invasion in pancreatic cancer.. Am J Pathol.

[40] Carstens RP, Eaton JV, Krigman HR, Walther PJ, Garcia-Blanco MA (1997). Alternative splicing of fibroblast growth factor receptor 2 (FGF-R2) in human prostate cancer.. Oncogene.

[41] Zhang Y, Wang H, Toratani S, Sato JD, Kan M (2001). Growth inhibition by keratinocyte growth factor receptor of human salivary adenocarcinoma cells through induction of differentiation and apoptosis.. Proc Natl Acad Sci U S A.

[42] Koos RD, Banks PK, Inkster SE, Yue W, Brodie AM (1993). Detection of aromatase and keratinocyte growth factor expression in breast tumors using reverse transcription-polymerase chain reaction.. J Steroid Biochem Mol Biol.

[43] Bansal GS, Cox HC, Marsh S, Gomm JJ, Yiangou C (1997). Expression of keratinocyte growth factor and its receptor in human breast cancer.. Br J Cancer.

[44] Liu N, Furukawa T, Kobari M, Tsao MS (1998). Comparative phenotypic studies of duct epithelial cell lines derived from normal human pancreas and pancreatic carcinoma.. Am J Pathol.

[45] Lotti LV, Rotolo S, Francescangeli F, Frati L, Torrisi MR (2007). AKT and MAPK signaling in KGF-treated and UVB-exposed human epidermal cells.. J Cell Physiol.

[46] Easton DF, Pooley KA, Dunning AM, Pharoah PD, Thompson D (2007). Genome-wide association study identifies novel breast cancer susceptibility loci.. Nature.

[47] Hunter DJ, Kraft P, Jacobs KB, Cox DG, Yeager M (2007). A genome-wide association study identifies alleles in FGFR2 associated with risk of sporadic postmenopausal breast cancer.. Nat Genet.

[48] Panici PB, Bellati F, Boni T, Francescangeli F, Frati L (2007). Vaginoplasty using autologous in vitro cultured vaginal tissue in a patient with Mayer-von-Rokitansky-Kuster-Hauser syndrome.. Hum Reprod.

[49] Tuschl T, Zamore PD, Lehmann R, Bartel DP, Sharp PA (1999). Targeted mRNA degradation by double-stranded RNA in vitro.. Genes Dev.

[50] Pfaffl MW (2001). A new mathematical model for relative quantification in real-time RT-PCR.. Nucleic Acids Res.

[51] Gillies RJ, Didier N, Denton M (1986). Determination of cell number in monolayer cultures.. Anal Biochem.

